# Ekbom Syndrome With Folie à Deux – an Examination of Nature vs Nurture Through a Case Study

**DOI:** 10.1192/bjo.2025.10718

**Published:** 2025-06-20

**Authors:** Aditi Dawar, Shaimaa Aboelenien

**Affiliations:** EPUNFT, Rochford, United Kingdom

## Abstract

**Aims:** Delusional parasitosis (DP), or Ekbom syndrome, is a rare psychiatric disorder in which individuals falsely believe they are infested with parasites. When shared by another person, it is classified as folie à deux (shared psychotic disorder). This case study explores a unique DP case within a close relationship, examining clinical presentation, potential causes, and treatment outcomes.

**Methods:** A 64-year-old woman sought mental health services, convinced she had fatally infested her 26-year-old neurodivergent son with scabies and transferred her heart disease to him. She had believed for years that she had a scabies infestation, a delusion shared by her mother, who had recently passed away at 89. Multiple dermatology consultations ruled out any infestation, yet she continued self-treating with bleach, essential oils, borax, and horse skin infection chemicals. She also took excessive baths, scrubbing herself vigorously.

Additionally, she was convinced she had heart disease and past cancers, though no medical evidence supported these claims. She exhibited significant anxiety and distress but denied perceptual disturbances and lacked insight into her condition. Treatment was initiated with a combination of an antipsychotic and an antidepressant, leading to a gradual reduction in delusional intensity and increased engagement with mental health services. Psychological support was also provided.

**Results:** DP is challenging to treat, particularly when reinforced by family members, as seen in this case. The patient’s condition worsened following her mother’s death. However, a multidisciplinary approach is essential to enhance engagement and compliance, which are crucial for a positive prognosis.

**Conclusion:** Understanding such cases is vital for accurate diagnosis and effective intervention, especially given the reinforcing nature of shared delusions and the persistent nature of DP.

